# Broadband integrated optic polarization splitters by incorporating polarization mode extracting waveguide

**DOI:** 10.1038/s41598-017-05324-x

**Published:** 2017-07-06

**Authors:** Guanghao Huang, Tae-Hyun Park, Min-Cheol Oh

**Affiliations:** Department of Electronics Engineering, Pusan National University, Busan, 46241 Korea

## Abstract

A compact integrated optic polarization splitter is highly anticipated for polarization multiplexed optical communications, dual polarization optical sensors, single photon quantum signal processing, etc. In this work, we propose and demonstrate a polarization mode extracting device using a highly birefringent crosslinked liquid crystal polymer, reactive mesogen. The device includes the birefringent material inserted into a Y-branch optical waveguide to extract the TE polarized mode. A polarization splitting ratio of 27 dB was obtained, and a crosstalk of less than −25 dB was maintained for a wavelength range of 1500~1600 nm. The device exhibited good thermal stability at 100 °C for 12 hours. The broadband operating characteristic is a unique advantage of the proposed mode extracting polarization splitter.

## Introduction

The polarization splitter is an indispensable optical device for polarization multiplexed optical communication^1, 2^, dual polarization optical sensor^3, 4^, and single photon quantum signal processing^5^, etc. Especially, for the Bell state measurement in quantum key distribution, compact integrated-optic polarization splitters are highly anticipated^6–8^.

Polarization splitters have been demonstrated based on various optical materials such as silicon, silica, and polymers. The large waveguide contrast in silicon photonic devices enables small device dimension for high density integration. The polarization splitters in silicon were demonstrated by using geometry-induced birefringence in a directional coupler waveguide^9, 10^, polarization sensitive slot waveguide directional coupler^11^, polarization-dependent critical guiding condition of an asymmetrical directional coupler^12^, diffraction grating coupler with a polarization sensitive diffraction angle^13^, asymmetric Mach-Zehnder structure^14, 15^, and a nanophotonic directional coupler controlling the interaction of guided resonant modes^16^. However, due to the high index contrast of the silicon, device performance is sensitive to dimensional changes in the waveguide, which worsens manufacturing tolerances.

In a silica polarization splitter, the amorphous silicon film was deposited on one arm of the Mach–Zehnder interferometer and stressed the waveguide to impart polarization-dependent phase delay through the stress-optical effect^17, 18^. An additional trimming process was required to accurately adjust the relative phase difference. A metal loaded directional coupler structure^19^ and direct laser written birefringent directional coupler^20^ were also proposed.

Polymer has the advantage in controlling the birefringence of the optical waveguide due to the variety of molecular structures^21^. Depending on the length of the oligomer molecule, the crosslinked polymer layer coated on a substrate possesses different optical birefringence. Polymer optical waveguide polarization splitter has been demonstrated using a variety of birefringent materials such as poled polymers^22, 23^, perfluorinated birefringent polymers^24, 25^ and reactive mesogenic liquid crystal materials^26^. The low splitting ratio and instability of the birefringence imposed by the prior device was a problem to be solved. Reflective polarizing splitter based on total reflection of TE polarized light has been proposed to have excellent polarization splitting ratio and temperature stability^26^. However, the reflective polarizing splitter requires a long device length due to the transition between the channel and the planar waveguide.

In this work, we propose a compact polarization mode extracting device utilizing highly birefringent polymer. It has a tiny mode converter with a taper that is effective only for TE polarized light, and then the TE polarized mode is extracted by another path. The proposed mode extracting polarization splitter has a large fabrication tolerance because it is not based on the polarization dependent phase change that is hard to reproduce. Additionally, the polarization extracting device has no dependence on the wavelength and inherently guarantees broadband operation.

## Results

### Operating principle of polarization extractor

Polymers have various merits that can control optical birefringence according to their molecular structure and can be fabricated on various substrates by using a simple spin coating process. Reactive Mesogen (RM), a kind of liquid crystal, forms an optically uniaxial thin film with an optical axis in the plane direction^27^. According to the prism coupling refractive index measurement, the RM film exhibited refractive index values of 1.6457 and 1.5205 for TE and TM polarized light, respectively. However, we found the RM could have TM refractive index as high as 1.536 during the experiment of the polarization splitter device based on total internal reflection interface^26^. A low birefringence polymer named CO-polymer produced by ChemOptics Co. was also used for device fabrication. It has a refractive index of 1.540 and a small birefringence of 0.001.

**Figure 1:**
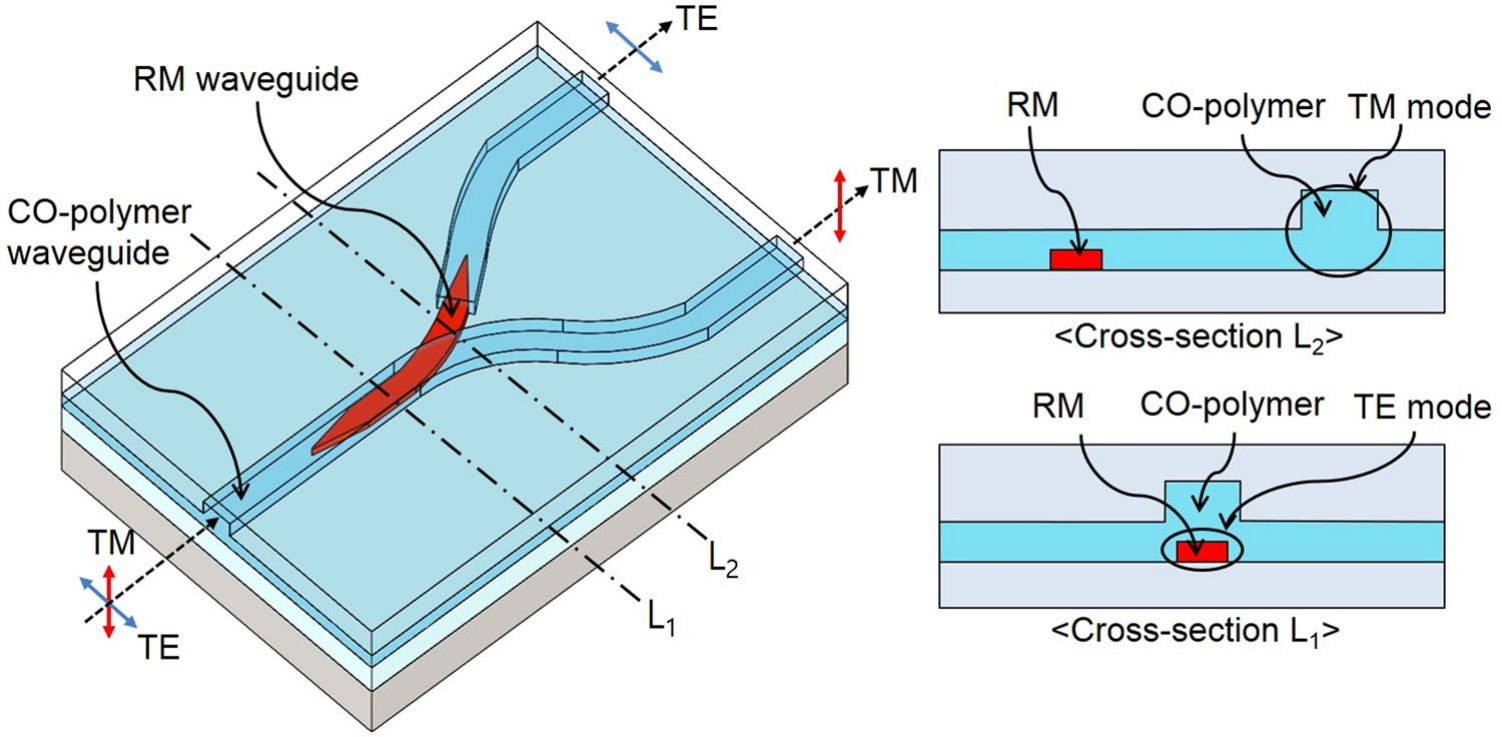
Schematic diagram of the polarization splitter consisting of a taper for extracting TE polarized light, and a Y-branch type CO-polymer waveguide.

The polarization splitter proposed in this work incorporates the birefringence material RM inserted within the Y-branch optical waveguide to extract TE polarized light. As shown in Fig. 1, in the case of TM polarized light, the CO-polymer waveguide has a relatively higher refractive index than the RM waveguide, so that the light follows the CO-polymer waveguide. However, for TE polarization, RM has the higher refractive index, then the TE polarized light is coupled into the RM waveguide through the taper structure. The RM waveguide is positioned to connect the gap portion so that the TE polarized light can pass through the disconnected branch of the CO waveguide.

### Characterization of the fabricated device

**Figure 2:**
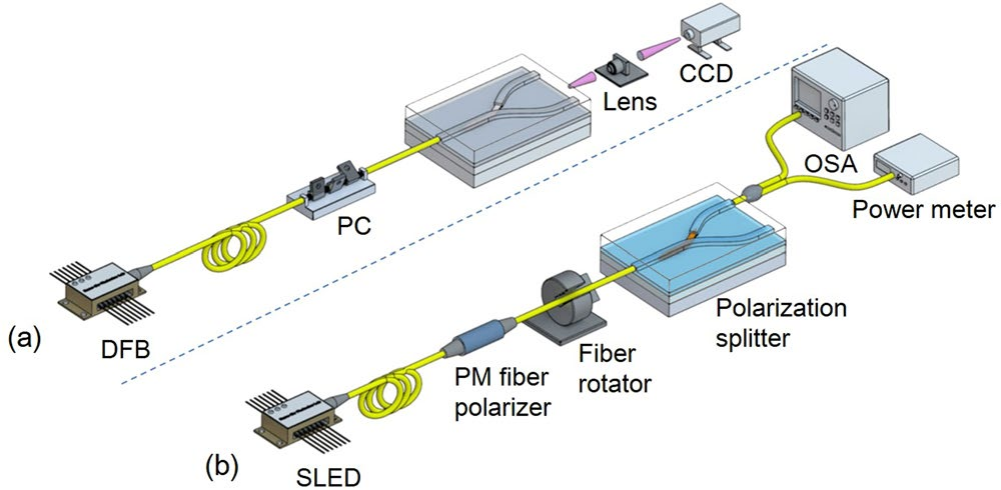
Integrated optic device characterization setup for (**a**) output mode image observation consisting of DFB laser, fiber-optic polarization controller (PC), polarization splitter, objective lens, and CCD image sensor, and (**b**) broadband spectral response measurement consisting of SLED light source, PM fiber polarizer, fiber rotation stage, polarization splitter device under test, optical spectrum analyzer and power meter.

**Figure 3:**
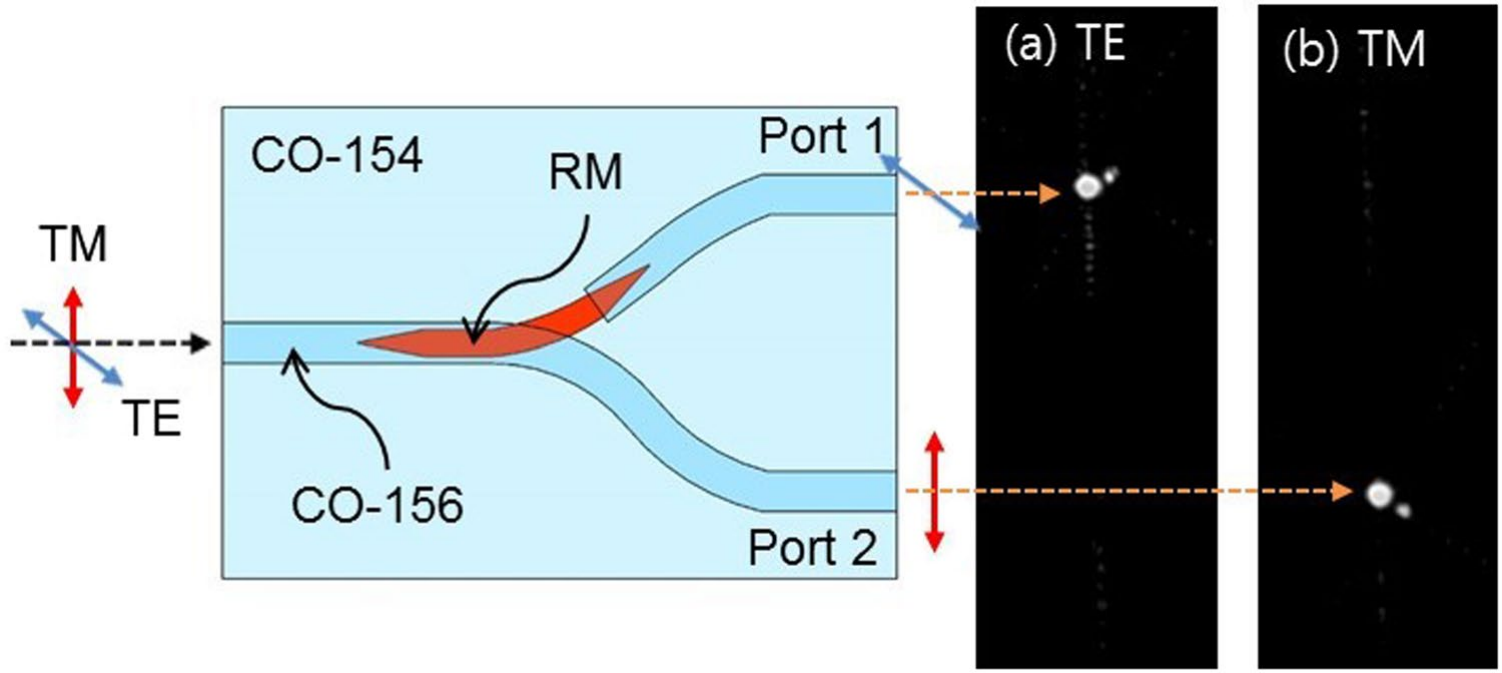
CCD images of the output modes for the inputs with (**a**) TE polarization, and (**b**) TM polarization, in which a crosstalk to the other output port is negligible.

To characterize the fabricated polarization splitter, a DFB laser with a center wavelength of 1550 nm was used, and TE/TM polarized light was selectively excited using a fiber-optic polarization controller. The measurement setup is shown in Fig. 2(a). At first, the output light from the device was observed by using a charge-coupled device (CCD). As can be seen in Fig. 3, TE polarized light excited in the CO-polymer waveguide was coupled into the RM waveguide and went out to the port 1, while TM polarized light was directly going out to port 2 regardless of the RM waveguide. The output power was measured by aligning a single mode fiber to the waveguide output. For TE input, the insertion losses at port 1 and port 2 were 4.7 dB and 32.0 dB, respectively, and for TM input, they were 31.3 dB and 4.3 dB, respectively. The crosstalk for each polarization was about −27 dB.

The main causes of the loss are the propagation loss, the coupling loss, and the mode conversion loss by the taper. The propagation losses for the CO-polymer waveguide and the RM waveguide measured through the cut-back method with 1550 nm light source were 1.1 and 3.2 dB/cm, respectively. The coupling loss between single mode fiber and the CO-polymer waveguide was 1.7 dB/facet in the cut-back measurement. In the case of TM polarized light, the insertion loss of 4.3 dB includes contributions due to 0.8 dB of propagation loss in 0.72-cm long CO-polymer waveguide, 3.4 dB of fiber coupling loss and 0.1 dB of additional loss due to the perturbation by RM waveguide. For TE polarized light, the loss through the RM-waveguide of 0.28 cm length is considered to be 0.9 dB, and the total propagation loss is 1.4 dB. Then, by including the fiber coupling loss of 3.4 dB, the insertion loss comes close to the measured value of 4.7 dB. From this loss factorization, we conclude that the taper of the RM-waveguide does not make an important contribution as expected in the BPM simulation results.

**Figure 4:**
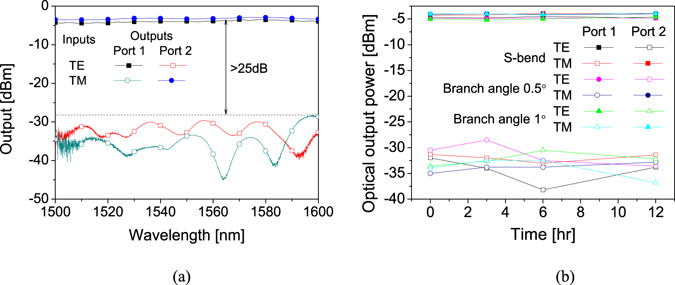
(**a**) Measured wavelength dependence over the wavelength range of 1500~1600 nm, and a crosstalk of less than −20 dB was found, and (**b**) Thermal stability of the fabricated polarization splitter with various branch structures.

To increase the communication capacity of the WDM optical communication system, different wavelengths over a wide range are used. Therefore, the operating bandwidth characteristic of the polarization splitter is important. For a broadband spectral response measurement, a SLED light source with a 3-dB bandwidth of 45.6 nm and a center wavelength of 1559.7 nm was prepared. Due to the limited bandwidth of fiber-optic polarization controller, the controller was replaced by a fiber-optic polarizer and a rotatable polarization maintaining (PM) fiber as shown in Fig. 2(b). By adjusting the angle of PM fiber, it was possible to adjust the polarization with a polarization extinction ratio of 30 dB in the wavelength range of 1500 to 1600 nm. As shown in the measurement results of Fig. 4(a), it was confirmed that the device provided a crosstalk of less than -25 dB over a wide wavelength band.

In order to confirm the performance of polymer device is reliable at high temperature, 100 °C oven was used for temperature stability test. Several devices with different branch structure fabricated on the same substrate were examined. The Y-branches were formed as an s-bend and linear branches with branch angles of 0.5° and 1°. The comparison of the measured results is shown in Fig. 4(b). After 12 hours, the insertion loss and the crosstalk of the device were not changed.

## Discussion

The polarization mode extracting structure is fabricated using a highly birefringent polymer and has been successfully applied to realize a broadband polarization splitter device. The birefringent polymer waveguide taper has been used to extract the TE polarized mode and directs it to another path. The device constructed with two different kinds of polymers exhibited a high polarization extinction ratio at a bandwidth of 100 nm, which was not demonstrated previously. The proposed device can be further operable for 1300 nm as long as the waveguide is designed to satisfy a single mode condition, since the operating principle has no wavelength dependence.

The insertion loss of the device was factorized in terms of propagation loss, coupling loss, and mode conversion loss in the taper. The current device has relatively large propagation loss, however it could be reduced by developing a highly fluorinated polymer with a sufficient birefringence. Fluorinated polymer waveguide device typically exhibits propagation loss less than 0.2 dB/cm. The coupling loss could also be reduced by enlarging the dimension of over-sized rib waveguide or by incorporating a small core optical fiber. Polymer has a refractive index close to that of silica optical fiber, therefore the Fresnel loss is negligible, which is hard to achieve in silicon photonics. Through a careful mode size matching, the coupling loss in commercial polymer waveguide devices have becomes 0.1~0.2 dB. The excess loss by the birefringent polymer taper was found to be very small as confirmed in this experiment. Conclusively, the insertion loss of the proposed polarization extracting device could be less than 0.5 dB in total.

The fabrication tolerance of integrated optic device depends on the allowable variation of the waveguide dimensions due to the photolithograph and the dry etching of polymer. The critical dimension of the proposed device is the blunted tip width at the end of the taper, which is appearing after the dry etching. As we have confirmed in the simulation and the experimental results, the blunt width of less than 0.5 μm has no effect on the device performance, which is not difficult to achieve in a typical photolithography process. Alignment between the two waveguides is another factor affecting the manufacturing yield. The adiabatic mode coupling in the birefringent waveguide taper has a misalignment tolerance as large as 1.0 μm, which increases fabrication repeatability. Compared to the previous polarization splitters based on polarization dependent phase change, the proposed polarization extraction device has a much higher production yield with good uniformity, which is important for constructing large scale integrated optics composed of many different types of waveguide devices.

## Methods

### Material preparation

For a highly birefringent polymer material, a reactive mesogen (RM) monomer solution (RMS03-013) with proper birefringent refractive indices was purchased from Merck Performance Materials Ltd. It was directly spin coated on the wafer. CO-series polymers for a core and cladding layers of the low birefringence waveguide with a refractive index of 1.54 and 1.52 were purchased from ChemOptics Co. The refractive index of core layer CO polymer was chosen to be close to that of TM index of RM.

### Design of the polarization extracting taper structure

**Figure 5:**
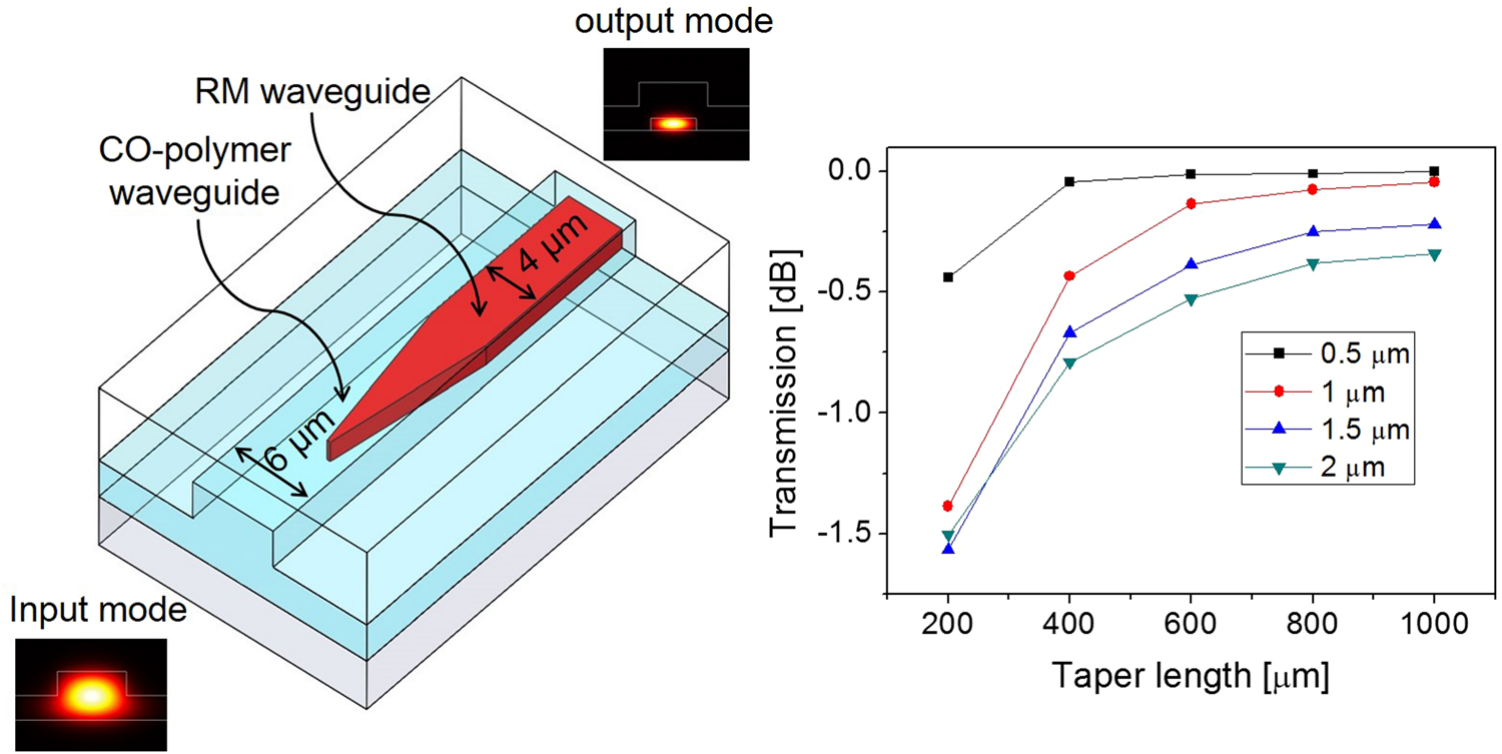
BPM simulation results for the TE mode conversion efficiency of the tapered RM waveguide, where the coupling loss less than 0.2 dB is available for the longer taper.

In a beam propagation method design, the widths of the CO-polymer and the RM waveguide were 6 and 4 μm, respectively. As shown in Fig. 5, the input mode confined in the CO-polymer waveguide was converted to an output mode that was well-confined in the RM waveguide through the taper. The final output power was obtained by calculating the mode field overlap integral. The mode conversion loss could be negligible if the taper was longer than 800 μm.

In this experiment, the waveguide is designed as an oversized rib structure, where a core layer remains at the side of waveguide core. As a result, the effective index difference between the core and the lateral cladding area is reduced, and then only a single mode satisfies the guiding condition even for the large core dimension. The oversized rib waveguide has been used to produce a single mode waveguide in a high contrast waveguide based on silicon on insulator^28^, and an electro-optic polymer^29^.

### Fabrication of the polymeric integrated optic device

**Figure 6:**
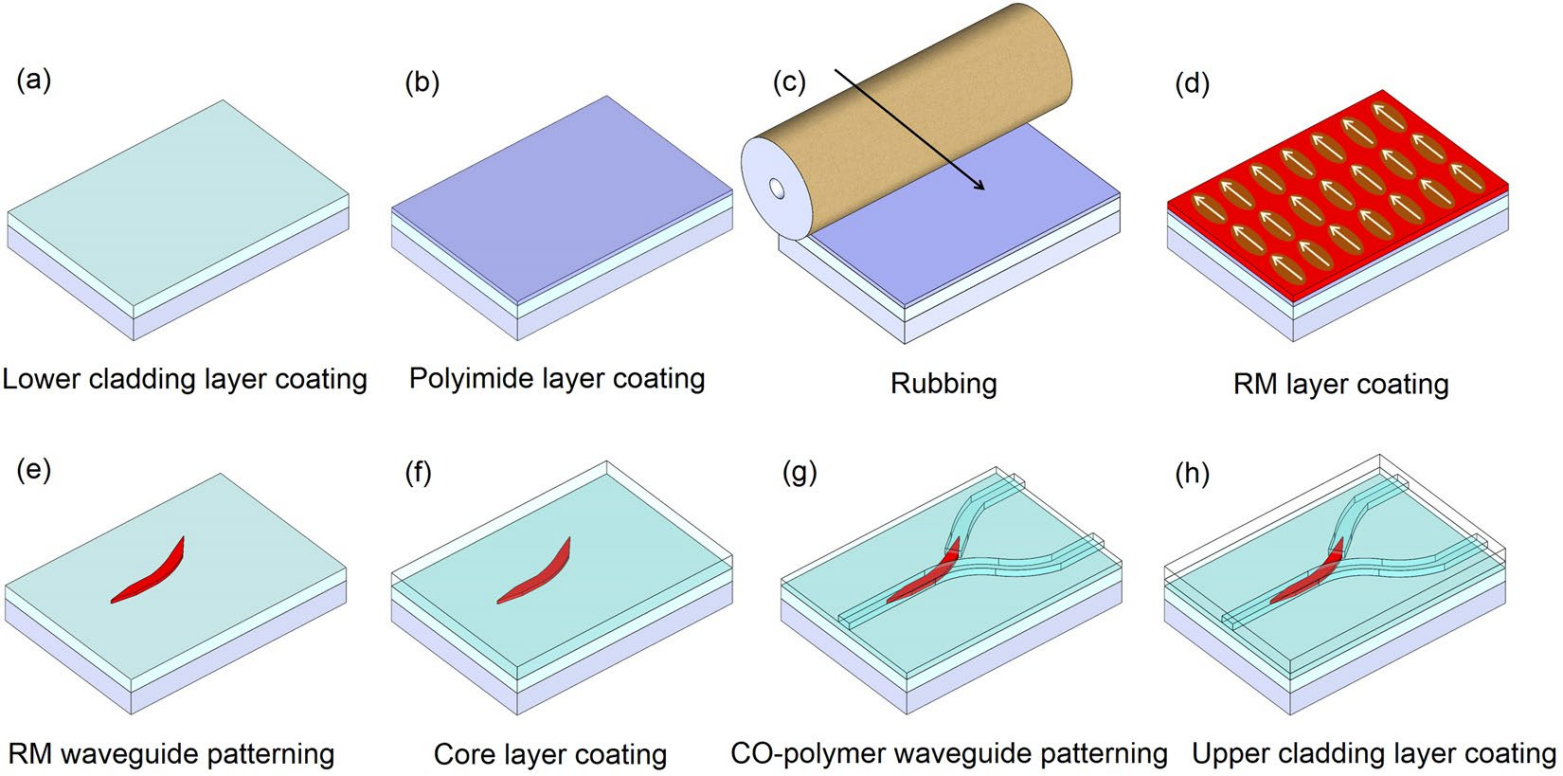
Schematic fabrication procedure of the mode extracting polarization splitter.

**Figure 7:**
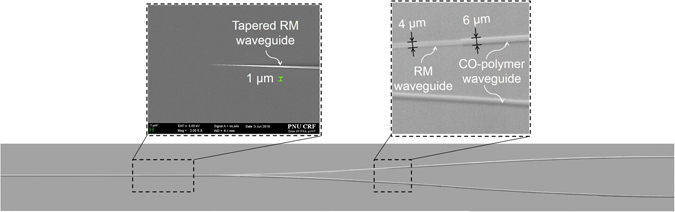
Microphotograph of the polarization splitting Y-branch waveguide, and SEM image of the tapered RM waveguide where the width is gradually decreasing to much less than 1 μm.

In the process of fabricating the proposed polarizer splitter using a RM material having a large birefringence and a CO-polymer having a low birefringence, a general manufacturing process such as spin coating, UV curing and photolithography, and a polyimide thin film Rubbing process is required as shown in Fig. 6. The lower cladding layer with a thickness of 8 μm was formed using CO-polymer with a refractive index of 1.520. A polyimide thin film with a thickness of 0.2 μm was formed thereon and rubbed in a specific direction using a velvet roller^30^. Then the RM material was coated and the molecules were aligned in the direction of rubbing. This results in an increase in the refractive index for TE polarized light parallel to the plane and a decrease in the refractive index for TM polarization. Then it was cured by UV of 9 mW/cm^2^ for 6 min. A photoresist was coated on the RM layer to form an RM waveguide pattern through photolithography and an oxygen plasma etching. For a plasma power density of 0.82 mW/cm^2^ and a chamber pressure of 50 mTorr, the etch rate of RM was 0.48 μm/min. The RM waveguide was formed with a width of 4 μm and a thickness of 1.1 μm. After oxygen plasma etching, the SEM image of the tapered RM waveguide was taken as shown in Fig. 7.

To form a low birefringence waveguide, CO-polymer with a refractive index of 1.540 was coated to a thickness of 4.2 μm and a Y-branch pattern was formed by photolithography. A rib waveguide with a width of 6 µm and a lateral core with a thickness of 1.8 µm was formed by using oxygen plasma etching process. Then a CO-polymer material for cladding with a refractive index of 1.520 was coated to a thickness of 8 µm to finish the optical waveguide.
